# 751. Decoding a Decade: Temporal Trends of MIC Distribution of Antifungals Against *Cryptococcus* species From a Reference Laboratory

**DOI:** 10.1093/ofid/ofac492.036

**Published:** 2022-12-15

**Authors:** Eloy E Ordaya, Omar M Abu Saleh, Paschalis Vergidis, Sharon Deml, Nancy Wengenack, Madiha Fida

**Affiliations:** Mayo Clinic, Rochester, Minnesota; Division of Public Health, Infectious Diseases, and Occupational Medicine, Department of Medicine, Mayo Clinic, Rochester, Minnesota; Mayo Clinic, Rochester, Minnesota; Mayo Clinic, Rochester, Minnesota; Mayo Clinic, Rochester, Minnesota; Mayo Clinic, Rochester, Minnesota

## Abstract

**Background:**

There are not established clinical breakpoints (CBP) for antifungals against *Cryptococcus* species; However, the analysis of their MICs using epidemiological cut-off values (ECVs) can help to distinguish wild-type (WT) isolates from non-WT strains that may harbor intrinsic or acquired resistance to antifungals. Herein, we analyze the MIC distribution of antifungals against *Cryptococcus* spp. isolated over the last decade at our reference laboratory.

**Methods:**

We conducted a retrospective evaluation of antifungals MICs for *Cryptococcus neoformans* and *Cryptococcus gattii* complex isolates from various sources that were processed at the Mayo Clinic Laboratory from November 18, 2011 to June 7, 2021. Antifungal susceptibility testing was performed by the Clinical & Laboratory Standards Institute (CLSI) standards (CLSI M59-ED3:2020). We used recommended ECVs to define if the isolates were WT or non-WT, and described the change in the percentage of WT isolates over time.

**Results:**

During the study period, *C. neoformans* was isolated from 632 samples collected from 533 patients. Sample sources included cultures from blood (n=301), cerebrospinal fluid (n=230), respiratory (n=71), and other sources (n=30). The overall percentage of WT isolates for amphotericin B (AMB), 5-flucytosine, and fluconazole were 77%, 98% and 91% respectively with no significant difference based on various sources (Table). We noticed an overall increase in MICs for AMB, with 100% WT isolates in 2011 compared to 79% in 2021. No such increase was seen for fluconazole or 5-Flucytosine (Figure). Irrespective of the source or the year of isolation, WT strains for itraconazole, voriconazole, and posaconazole were consistently > 93%. *C. gattii* was isolated from 16 samples, and 94% were WT strains for AMB and 100% for the other antifungals.

CLSI, Clinical and Laboratory Standard Institute; CSF, cerebrospinal fluid; ECVs, epidemiologic cut-off values.

AMB, amphotericin B; FLU, fluconazole; 5FC, 5-flucytosine.

**Conclusion:**

In the last decade, we have observed an overall increase in MICs of AMB for *C. neoformans*. The number of WT strains for other antifungals was high and not significantly changed during the study frame. Further studies are needed to identify the clinical implications of these findings.

**Disclosures:**

**Paschalis Vergidis, MD**, AbbVie: DSMB|Cidara: Grant/Research Support|Scynexis: Grant/Research Support.

